# MiR-199a-5p Decreases Esophageal Cancer Cell Proliferation Partially through Repression of Jun-B

**DOI:** 10.3390/cancers15194811

**Published:** 2023-09-30

**Authors:** Pornima Phatak, Mohan E. Tulapurkar, Whitney M. Burrows, James M. Donahue

**Affiliations:** 1 Birmingham Veterans Affairs Health Care System, Birmingham, AL 35233, USA; 2 Department of Surgery, University of Alabama at Birmingham, Birmingham, AL 35233, USA; 3 Department of Medicine, University of Maryland School of Medicine, Baltimore, MD 21201, USA; mtulapurkar@som.umaryland.edu; 4 Department of Surgery Thoracic Medicine and Surgery, Lewis Katz School of Medicine, Temple University, Philadelphia, PA 19140, USA

**Keywords:** miR-199a-5p, Jun-B, esophageal cancer, proliferation

## Abstract

**Simple Summary:**

The expression of specific microRNAs may be significantly altered in different kinds of cancers. MiR-199a-5p has been shown to be downregulated in multiple malignancies and function as a tumor suppressor. We have previously shown that miR-199a-5p is markedly downregulated in esophageal squamous cancer cell lines compared to esophageal epithelial cells. MiR-199a-5p is predicted to interact directly with Jun-B mRNA, an important component of the AP1 transcription factor, with high affinity. The aim of our study was to determine expression of Jun-B in esophageal cancer cells as well as to investigate the interaction between miR-199a-5p and Jun-B in these cells and to characterize the functional implications of this interaction.

**Abstract:**

MicroRNA (miR)-199a-5p has been shown to function as a tumor suppressor in some malignancies but its role in esophageal cancer is poorly understood. To further explore its role in esophageal cancer, we sought to investigate the interaction between miR-199a-5p and Jun-B, an important component of the AP1 transcription factor, which contains a potential binding site for miR-199a-5p in its mRNA. We found that levels of miR-199a-5p are reduced in both human esophageal cancer specimens and in multiple esophageal cancer cell lines compared to esophageal epithelial cells. Jun-B expression is correspondingly elevated in these tumor specimens and in several cell lines compared to esophageal epithelial cells. Jun-B mRNA expression and stability, as well as protein expression, are markedly decreased following miR-199a-5p overexpression. A direct interaction between miR-199a-5p and Jun-B mRNA was confirmed by a biotinylated RNA-pull down assay and luciferase reporter constructs. Either forced expression of miR-199a-5p or Jun-B silencing led to a significant decrease in cellular proliferation as well as in AP-1 promoter activity. Our results provide evidence that miR-199a-5p functions as a tumor suppressor in esophageal cancer cells by regulating cellular proliferation, partially through repression of Jun B.

## 1. Introduction

The incidence of esophageal cancer continues to increase and it is now the sixth leading cause of cancer-related death in the world [1]. Based on current incidence rates, the expected number of annual deaths worldwide from esophageal cancer is expected to increase by approximately 60% from 544,100 to 880,000 by 2040 [2]. Overall, five-year survival remains dismal at approximately 20% despite improved pathologic complete response rates with newer neo-adjuvant strategies and the recently established efficacy of Nivolumab, a PD-1 inhibitor, in the adjuvant setting for patients with persistent disease following tri-modality therapy [3,4,5]. Given the rapidly rising prevalence of this deadly disease, increased efforts aimed at understanding the molecular mechanisms underlying the development and progression of esophageal cancer are urgently required. Despite a recent thorough analysis of the esophageal cancer genome, currently no targetable driver mutations have been shown to demonstrate clinical efficacy in the treatment of esophageal cancer [6,7]. As sequencing alone does not appear to be sufficient to identify novel therapeutic and predictive targets, alternative molecular strategies are required to improve outcomes for esophageal cancer patients. 

MicroRNAs (miRs), which are short and non-coding RNAs that function as critical post-transcriptional regulators of gene expression in cancer cells, hold great potential as both biomarkers and therapeutic targets [8]. As is the case in most cancers, numerous studies have described the ability of multiple individual miRs to regulate the expression of several targets with oncogenic or tumor suppressive functions in esophageal cancer cells [9,10]. More importantly, a germline knockout of miR-31 was recently shown to be able to eliminate the development of esophageal squamous cell cancer in a zinc-deficient rat model, demonstrating the importance of miRs in esophageal carcinogenesis [11]. 

When choosing which miR to study for potential clinical applicability, several factors must be considered, including differences in expression between normal and malignant cells and the ability to regulate multiple targets. In a previous study comparing global miR expression between a human esophageal epithelial cell line (hESO) and the human esophageal squamous cell cancer lines TE7 and TE10 using array analysis, we found that miR-199a-5p was the most downregulated miR in the cancer cell lines [12]. In this study, the expression of miR-199a-5p was 4.4 log orders decreased in both TE7 and TE10 cells compared to hESO cells. In a separate analysis, we showed that miR-199a-5p overexpression could reduce proliferation in TE7 cells by decreasing expression of mitogen-activated protein kinase kinase kinase-11 (MAP3K11) [13]. 

The purpose of this study was to identify an additional oncogenic target regulated by miR-199a-5p in esophageal cancer cells in order to better establish its potential clinical relevance. Using miR-target prediction programs (miRDB and Target Scan Human 7.2), we found that miR-199a-5p is predicted to bind the mRNA encoding the Jun-B protein with high affinity. Along with c-JUN, Jun-D, and the Fos family proteins, Jun-B is a major component of the AP-1 transcription factor [14]. Although initially thought to exert a growth inhibiting function through antagonizing the effects of c-Jun, Jun-B has been shown to exert growth promoting effects that enhance neoplastic transformation is some tissues [15]. The expression, regulation, and function of Jun-B in esophageal cancer is poorly described. In this study, we have assessed miR-199a-5p and Jun-B expression in esophageal cancer cell lines and in human esophageal cancer specimens. Characterization of the interaction between miR-199a-5p and Jun-B mRNA was performed by assaying direct binding and by the use of functional assays including proliferation and colony formation in esophageal cancer cells. 

## 2. Materials and Methods

### 2.1. Tissue Culture

The human esophageal squamous cancer cell line TE7 was obtained and maintained in RPMI medium as previously described [12]. The human esophageal adenocarcinoma cancer cell lines OE-21 and SK-GT-4 were purchased from the European Collection of Authenticated Cell Culture (ECACC, England, UK) and were cultured in RPMI medium (Thermo-fisher, Carlsbad, CA, USA) supplemented with 10% heat-inactivated FBS (Invitrogen, Carlsbad, CA, USA). All cells were maintained in a 37 °C incubator with 5% CO_2_ humidified air.

### 2.2. MiR and JunB Plasmid Overexpression and Target mRNA Silencing

All transfections were conducted for 48 h unless specified otherwise. Briefly, 0.75–1 × 10^6^ cells were seeded in 60 mm (Thermo-fisher, Carlsbad, CA, USA) cell culture plates a day prior to transfection. For miR transfections, pre-miR-199a-5p (50 nM), or control miR (Ambion, Austin, TX, USA), was diluted in 500 μL Opti-MEM (Invitrogen, Carlsbad, CA, USA). Then, 5 μL of Lipofectamine RNAiMAX (Invitrogen, Carlsbad, CA, USA) per reaction was also diluted in 500 μL Opti-MEM. Diluted miRNA was mixed with RNAiMAX and was incubated at room temperature for 15 min. The complex was added to the cells in a final volume of 5 mL of fresh medium. For RNA silencing, a pool of four target specific Jun-B si-RNAs (50 pmol) (Dharmacon Inc, Lafayette, CO, USA) were used. For Jun-B overexpression, 2.5 μg of ORF expression clones for Jun-B (NM_002229.2) from GeneCopoeia, Rockville, MD, USA, was used. Lipofectamine 2000 transfection reagent ((Invitrogen, Carlsbad, CA, USA)) was used for plasmid overexpression experiments. 

### 2.3. cDNA Synthesis and Quantitative PCR

The total RNA concentration was measured on a Nanodrop1C (Thermo-fisher, Carlsbad, CA, USA). MiR reverse transcription (RT) was performed using a Taqman microRNA reverse transcription kit (Applied Bio systems, Foster City, CA, USA) and regular RT (Quantabio, Beverly, MA, USA) was conducted using a Quantabio qscript cDNA synthesis kit following the kit’s manual. For Real Time PCR (q-PCR), Taqman probes were used for targets Jun-B, miR-199a-5p, U6, and GAPDH (Applied Bio Systems, Foster City, CA, USA). All the PCR reactions were performed using the Quant Studio 3 system in triplicate. The levels of GAPDH were used to normalize levels of Jun-B in q-PCR samples. For miR experiments, normalization was accomplished using small nuclear RNA U6.

### 2.4. Human Tissue Specimens

Biopsies of human esophageal tumor as well as adjacent non-malignant epithelium with no gross evidence of tumor or Barrett’s esophagus were obtained at the time of esophagectomy from 13 patients as per an Institutional Review Board (IRB) approved protocol. Biopsy samples were snap-frozen in liquid nitrogen prior to processing. The tissues were ground to a fine powder using a mortar and pestle without allowing them to thaw. Tissue powder was homogenized using QIAshredder (Qiagen, Valencia, CA, USA) in 700 μL of lysis buffer and the total RNA was extracted using the miRNeasy Kit (Qiagen, Valencia, CA, USA) as per the manufacturer instruction manual.

### 2.5. Absolute Quantitative PCR

The absolute copy number for target mRNA and miR were measured using droplet digital PCR (Bio-Rad) as per the instruction manual. Droplet Digital PCR (ddPCR™) was performed using the QX200™ ddPCR™ system (Bio-Rad, Hercules, CA, USA). All reagents, disposables, and equipment are from Bio-Rad except the probes. The droplets were generated for each sample PCR reaction mixture using droplet generation oil as per the manufacturer’s instructions. Then, a C1000™ thermal cycler was used with cycling conditions of 95 °C for 10 min, followed by 40 cycles of 94 °C for 30 s/cycle, 60 °C for one minute, and finally 98 °C for 10 min. The plate was then transferred to the QX200™ Droplet reader and the data were analyzed using QuantaSoft™ Software version 1.7.

### 2.6. Western Blots 

Whole cell lysates (20 μg) were resolved on 10% SDS-PAGE gels (Bio-Rad Laboratories, Hercules, CA, USA) and immunoblots were performed as reported earlier [12]. The automated Western system JESS was used to run lysates from si-Jun-B transfections. Anti-human Jun-B (1:2000) antibody was purchased from Proteintech (Danvers, MA, USA), anti-GAPDH (1:2000) and horseradish peroxidase-conjugated anti-rabbit antibodies (1:3000) were purchased from Santa Cruz Biotechnology (Dallas, TX, USA). The signal intensity was quantified using Image Lab quantification software (Bio-Rad, Hercules, CA, USA) for conventional immunoblotting and a compass for simple Western software was used for automated Western.

### 2.7. MiR-Target Prediction

Two software programs, miRDB and Target Scan Human 7.2, were used to predict potential targets of miR-199a-5p. MiRDB is an online database for miRNA-target prediction and functional annotations. All the targets in miRDB were predicted by a bioinformatics tool, MirTarget, which was developed by analyzing thousands of miRNA-target interactions from high-throughput sequencing experiments. The target scan predicts biological targets of miRNAs by searching for the presence of conserved 8 mer, 7 mer, and 6 mer sites that match the seed region of each miRNA [16].

### 2.8. Transcription Decay Assay

A Jun-B mRNA degradation was assayed by measuring mRNA stability. TE7 cells in a 60 mm cell culture dish were transfected with pre-miR-199a-5p as described above for 24 h. Next, RPMI medium containing Actinomycin D (Sigma-Aldrich, St. Louis, MO, USA) at a final concentration of 4 μM was added for specified time points. The total RNA was isolated from each sample. RT and q-PCR were performed in triplicate as described above. The levels of Jun-B mRNA was normalized with the GAPDH levels.

### 2.9. Biotin-Labeled Pull-Down Assays

Biotinylated miR-199a-5p (5′ cccaguguucagacuaccuguuc 3′Bi) pull-down assays with target mRNA were performed as described earlier [12]. Biotinylated transcripts were synthesized from Dharmacon, Lafayette, CO. Overall, 1 × 10^6^ SK-GT-4 cells were transfected with biotin-labelled miR-199a-5p or control miR at a final concentration of 50 nM for 48 h. Cells were then scrapped and cell lysates were made. Whole cell lysates were incubated at 4 °C overnight with 50 μL/sample of streptavidin-Dyna beads (Invitrogen, Carlsbad, CA, USA) coated with yeast tRNA (10 mg/mL, Ambion, Austin, TX, USA). Next day, beads were washed thoroughly and RNA was isolated using TRIzol (Invitrogen) and the standard chloroform–isopropanol method and then subjected to PCR as explained above.

### 2.10. Luciferase Assasys

Two Jun-B (NM_002229.3) luciferase reporter constructs were generated. Primers for the constructs were designed using primer3 input software and SacI and XbaI enzyme sequences (bold sequences in the table) were added to them. Then, PCR-amplified (Go Green master mix, Promega, Madison, WI, USA) individual coding region (CR) and 3′UTR (untranslated region) insert fragments were sub-cloned into a SacI and Xba1 (New England Bio Labs, Ipswich, MA, USA) digested pmirGLO Dual-Luciferase miRNA target expression vector (Promega, Madison, WI, USA). The Jun-B 3′UTR construct, containing a deletion mutation of 8 bases (ACACTGGA) at the sequence of the predicted binding site, was generated using a site-directed mutagenesis kit (Agilent Technologies, Santa Clara, CA, USA) as per the kit’s manual. The primers for mutation were generated as per the kit’s requirement using Primer X software (Primer3Web 4.1.0, https://www.bioinformatics.org/primerx/, accessed on 15 July 2022). All primer sequences used to create these constructs are listed in Table 1. In total, 25 ng of wild type DNA were used for mutation PCR; PCR conditions were set as per the kit manual. The orientation and the sequence of the constructs were confirmed by restriction enzyme digestion and DNA sequencing (UAB genomics core lab). The AP1 promoter construct (E4111, Promega, Madison, WI, USA) was a kind gift from Dr. Ishwar S. Singh. The pGL4.44 [luc2P/AP1 RE/Hygro] vector contains six copies of an AP1 response element (AP1 RE) that drives transcription of the luciferase reporter gene luc2P [17]. For luciferase activity assays, 2 × 10^5^ SK-GT-4 cells/well were plated onto 12-well cell culture plates and the next day they were co-transfected as described above with luciferase reporter constructs (10 ng) and pre-miR-199a-5p (50 nM) for 36 h. Luciferase activity was measured using the dual luciferase reporter assay kit (Promega, Madison, WI, USA), as per the manufacturer’s protocol. Levels of firefly luciferase activity were normalized to Renilla luciferase activity. Cells transfected with control miR were considered to demonstrate 100% activity.

### 2.11. MTT Assay 

For the assay, 2500 cells per well were seeded in 96-well cell culture plates in 100 μL of medium. The next day, transfection was performed with pre-miR-199a-5p or si-Jun-B RNA. After 1–5 days, 20 μL (5 mg/mL in phosphate buffer saline) of 3-(4-5-dimehtylthiazol-2-yl)-2,5-diphenyltetrazolium bromide (MTT, Roche, Mannheim, Germany) was added for ~4 h at 37 °C. The medium was then removed and 200 μL/well of dimethyl sulfoxide was added to obtain dissolved purple formazan crystals. The optical density was measured at 540 nM. The optical density for control cells is considered as 100%.

### 2.12. Colony Formation Assay

In total, 0.75–1 × 10^6^ OE21, SKGT-4, and TE7 cells were plated in 60 mm dishes. The next day, transfection was performed with pre-miR-199a-5p (50 nM) or si-Jun B RNA (50 pM) as described in 2.2. Then, 48 h post transfection, cells were counted for the control and overexpressed or silenced; samples and 2000 cells/plate were re-seeded in 60 mm cell culture dish for 14 days. At that time, plates were stained with crystal violet solution (Sigma-Aldrich, St. Louis, MO, USA) and imaged. For staining, medium was removed and cells were washed thrice with 1 mL of TBST wash buffer and then 700 μL/plate crystal violet solution was added. Plates were shaken on a rocker shaker at room temperature slowly for an hour and then washed with 1 mL of deionized water four times and imaged on a BioRAD chemidoc imager.

### 2.13. Statistics 

**Figure 1:**
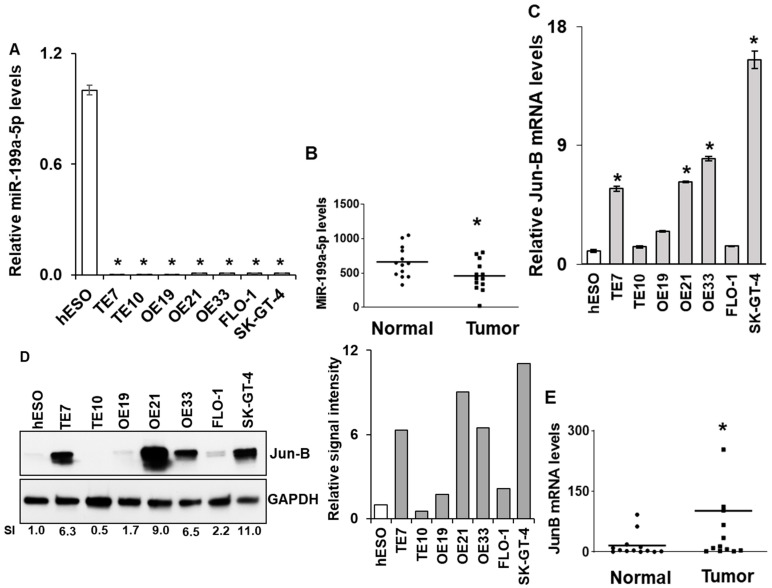
Baseline miR-199a-5p and Jun-B mRNA and protein expression levels in human esophageal cancer cell lines and specimens. Endogenous miR-199a-5p expression levels measured by real time PCR in (**A**) esophageal epithelial (hESO) and esophageal adenocarcinoma cell lines (OE19, OE21, OE33, FLO-1, and SK-GT-4). TE7 and TE10 squamous cancer cell lines are used as a positive control and in (**B**) human specimens. Relative levels of miR-199a-5p were normalized with small nuclear RNA U6. For cell lines, miR-199a-5p levels in tumor cells were compared with the level in the hESO cell line. Error bars represent ± S.D.; statistical significance based on a two-tailed Student’s *t*-test is indicated by * (*p* < 0.05). Baseline Jun-B mRNA levels in (**C**) human esophageal cell lines mentioned in (**A**) and in (**E**) human specimens. Relative Jun-B mRNA levels are normalized with GAPDH mRNA levels. For cell lines, Jun-B mRNA levels in tumor cells were compared with the level in the hESO cell line. (**D**) Endogenous Jun-B protein expression levels (left) in the esophageal cancer cell lines mentioned in (**A**). GAPDH was used as a loading control. Bar diagram (right) relative Jun-B signal intensity. S.I. = Relative Jun-B protein mean signal intensity. Signal intensity of the Jun-B is determined and is normalized by signal intensity of GAPDH. Relative signal intensity (SI) for Jun-B protein is calculated compared to hESO. Signal intensity was measured using Bio-RAD image lab software (version 6.1.0, Hercules, CA, USA). The uncropped blots and molecular weight markers are shown in Supplementary Figure S1.

Results are expressed as the means ± S.D from three independent experiments with a minimum of three replicates for each set of experiments. Data derived from multiple determinations were subjected to a two-tailed Student’s *t*-test and *p* values < 0.05 were considered significant. For human samples in Figure 1B,D, the Shapiro–Wilk test was used to confirm a normal distribution.

## 3. Results

### 3.1. Expression of miR-199a-5p and Jun-B in Esophageal Cancer Cell Lines and Human Esophageal Cancer Specimens

In order to expand on our previously published findings on miR-199a-5p levels in the esophageal squamous cell cancer lines TE7 and TE10, we measured the expression of miR-199a-5p in multiple esophageal adenocarcinoma cell lines by real-time PCR (q-PCR) [13]. As seen in Figure 1A, miR-199a-5p expression is significantly decreased compared to an esophageal epithelial cell line (hESO) in all five adenocarcinoma cell lines, with expression levels similar to those seen in the squamous cell cancer lines. In a similar fashion, we observed that miR-199a-5p expression was decreased in aggregate in esophageal tumor samples compared to matched esophageal epithelium taken from 13 esophageal cancer patients at the time of esophagectomy (*p* ≤ 0.05) (Figure 1B). Conversely, as seen in Figure 1C, levels of Jun-B mRNA are significantly increased in four of the cancer cell lines compared to hESO cells. The observed increase in Jun-B mRNA levels was associated with a marked enhancement in Jun-B protein expression in these cell lines compared to hESO cells assessed by Western blot (Figure 1D). This heterogeneous pattern in which Jun-B expression is not elevated in all cancer cell lines was also seen in comparing Jun-B mRNA levels between the esophageal cancer specimens and their matched esophageal epithelium (*p* ≤ 0.05) (Figure 1E).

### 3.2. Overexpression of miR-199a-5p Leads to Reductions in Jun-B mRNA and Protein Expression through Reduced Jun-B mRNA Stability

**Figure 2:**
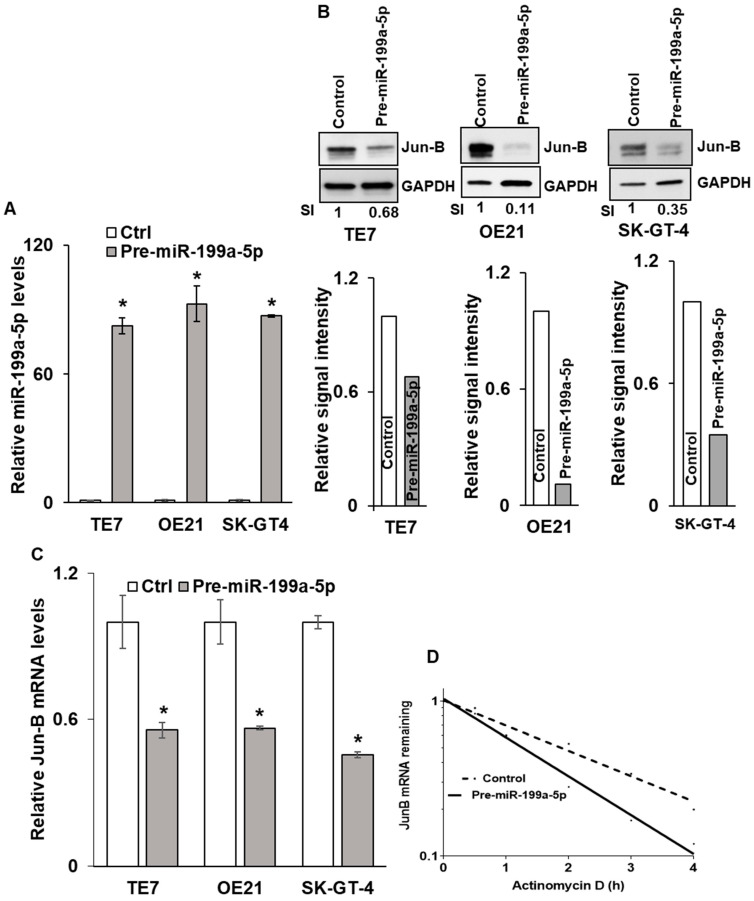
Effects of miR-199a-5p overexpression on Jun-B mRNA and protein. (**A**) Levels of miR-199a-5p after overexpressing miR-199a-5p in TE7, OE21, and SK-GT4 cells. Cells were transfected with pre-miR-199a-5p for 48 h. Total RNA was isolated and levels of miR-199a-5p were examined by q-PCR. Small nuclear U6 RNA was used as the control. Values are mean +/− SD from three independent experiments. (**B**) Changes in Jun-B protein expression levels in esophageal cancer cells following ectopic expression of miR-199a-5p. Whole-cell lysates were prepared for Western blotting 48 h after pre-miR-199a-5p transfection. Loading was assessed by GAPDH. (Bottom) Changes in signal intensity. Relative signal intensity in cells mentioned in (**B**), TE7 (left), OE21 (middle), and SK-GT-4 (right). Jun-B intensity was normalized with GAPDH intensity and relative levels were measured compared to the control for each cell line. Signal intensity for the control is set as 1 and intensity for over-expressed pre-miR-199a-5p TE7, OE21, and SK-GT-4 cells is 0.68, 0.11, and 0.35, respectively. (**C**) Changes in levels of Jun-B mRNA in TE7, OE21, and SK-GT-4 esophageal cancer cells following pre-miR-199a-5p ectopic expression. Cells were transfected with pre-miR-199a-5p for 48 h. Total RNA was extracted and levels of JUNB mRNA were measured by q-PCR. GAPDH was concurrently amplified to serve as an internal control. (**D**) Stability of Jun-B mRNA. Transcription of Jun-B mRNA was blocked post-pre-miR transfection by actinomycin D and levels of remaining mRNA were measured by PCR. The uncropped blots and molecular weight markers are shown in Supplementary Figure S2. The * indicates statistical significance.

Due to the fact that basal levels of miR-199a-5p are low in esophageal cancer cell lines, the transfection of pre-miR-199a-5p was performed in order to assess the effects on Jun-B protein expression (Figure 2A). The three cell lines with the highest levels of Jun-B protein expression (TE7, OE21, and SK-GT-4) were chosen for these experiments. Increased miR-199a-5p expression was associated with a marked decrease in Jun-B protein expression in all three cancer cell lines (Figure 2B). To investigate the mechanism by which miR-199a-5p regulates Jun-B protein expression, levels of Jun-B mRNA were assessed following transfection of pre-miR-199a-5p in TE7, OE21, and SK-GT-4 cells. As seen in Figure 2C, transfection of pre-miR-199a-5p was associated with a significant decrease in Jun-B mRNA levels in all three cancer cell lines. Next, we assessed the stability of Jun-B mRNA following transfection of pre-miR-199a-5p in TE7 cells. In these experiments, 24 h following transfection, cells are exposed to 4 μM Actinomycin D to prevent further transcription. The total cellular RNA was harvested at specified time points and levels of Jun-B mRNA were measured by q-PCR. As seen in Figure 2D, Jun-B mRNA is destabilized following the transfection of pre-miR-199a-5p, with an approximate 50% reduction in its half-life from 2.1 to 1.1 h.

### 3.3. MiR-199a-5p Binds to Jun-B mRNA

**Figure 3:**
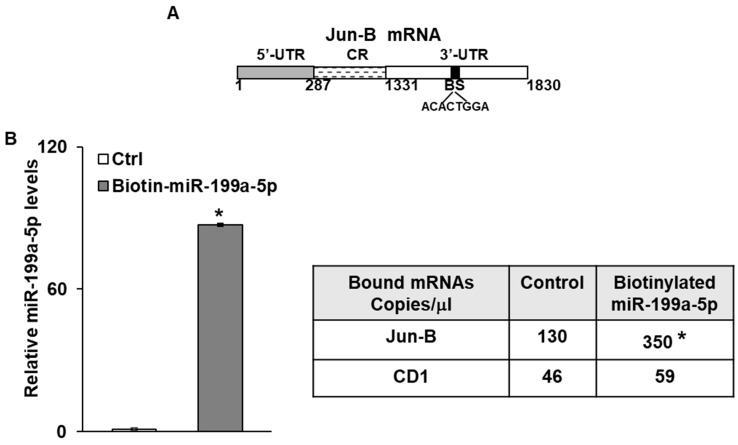

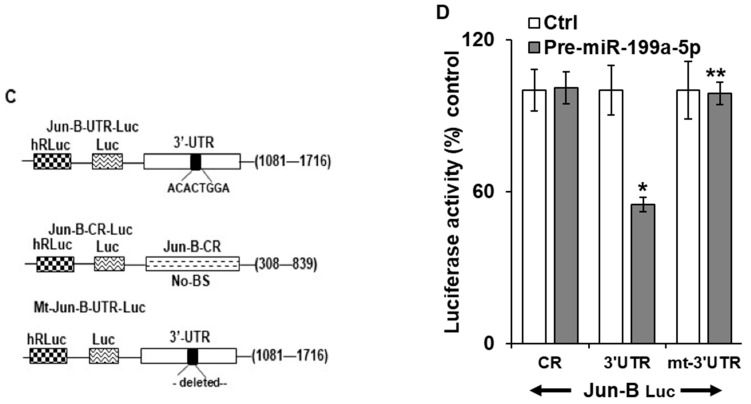
Interaction of miR-199a-5p with Jun-B mRNA. (**A**) Schematic diagram of Jun-B mRNA. (BS); (■) indicates the predicted binding sites for miR-199a-5p in 3′UTR. (**B**) Levels of biotinylated miR-199a-5p (5′ cccaguguucagacuaccuguuc 3′-Bi) after 48 h transfection as measured by q-PCR analysis. Levels were normalized with U6 RNA. Table Absolute levels (copy number) of Jun-B mRNA and Cyclin D1 mRNA in the materials pulled down by biotin-miR-199a-5p and control miR. Absolute levels were measured in copies/microliters using droplet digital PCR. (**C**) Schematic representation for Jun-B luciferase reporter constructs containing predicted binding site (top), no binding site (middle), or mutation, i.e., deletion of 8 bases in the predicted binding site for miR-199a-5p in 3′UTR (bottom). (**D**) Confirmation of Jun-B interaction with miR-199a-5p by luciferase assay. SK-GT-4 cells were co-transfected with pre-miR-199a-5p and constructs and changes in luciferase activity were measured. SD was presented in the form of error bars. * indicates significant miR-199a-5p binding with the 3′UTR of Jun-B mRNA compared to its CR. ** indicates a significant loss of this binding activity with the mutant construct compared to the WT 3′UTR (middle bar).

We next sought to determine whether miR-199a-5p directly interacted with Jun-B mRNA. As seen in Figure 3A, there is one predicted miR-199a-5p binding site in the 3′ untranslated region (UTR) of Jun-B mRNA. As a first step in the binding analysis, biotin-labelled miR-199a-5p or a biotin-labelled control miR were transfected into SK-GT-4 cells. Forty-eight hours later, cell lysates were prepared and exposed to streptavidin-coated beads. RNA was then isolated from the pull-down material and levels of Jun-B mRNA were measured by digital PCR. Levels of Jun-B mRNA were markedly elevated in the pull-down material harvested from the cells transfected with biotin-labelled miR-199a-5p compared to control transfection (Figure 3B). Importantly, there was no significant difference in the levels of Cyclin D1 mRNA, which served as a negative control in this experiment, in the pull down materials. 

In order to further verify this finding, three luciferase reporter constructs were created. In the first construct, a fragment of the Jun-B mRNA 3′ UTR containing the predicted miR-199a-5p binding site was inserted into the luciferase vector following PCR amplification. The second construct contained a portion of the Jun-B mRNA coding region (CR) which does not contain a predicted binding site for miR-199a-5p. In the third construct, the predicted binding sequence in the 3′ UTR was deleted and the resulting fragment was sub-cloned into the luciferase reporter vector (Figure 3C). Following co-transfection with pre-miR-199a-5p or control miR, there was approximately a 50% reduction in luciferase activity with the construct containing the predicted miR-199a-5p binding site, while no reduction in luciferase activity was observed with either the construct containing the fragment of the CR which does not contain a binding site or the construct containing the 3′UTR from which the predicted binding site was deleted (Figure 3D).

### 3.4. Overexpression of miR-199a-5p Reduces Esophageal Cancer Cell Proliferation through Downregulation of Jun-B

**Figure 4:**
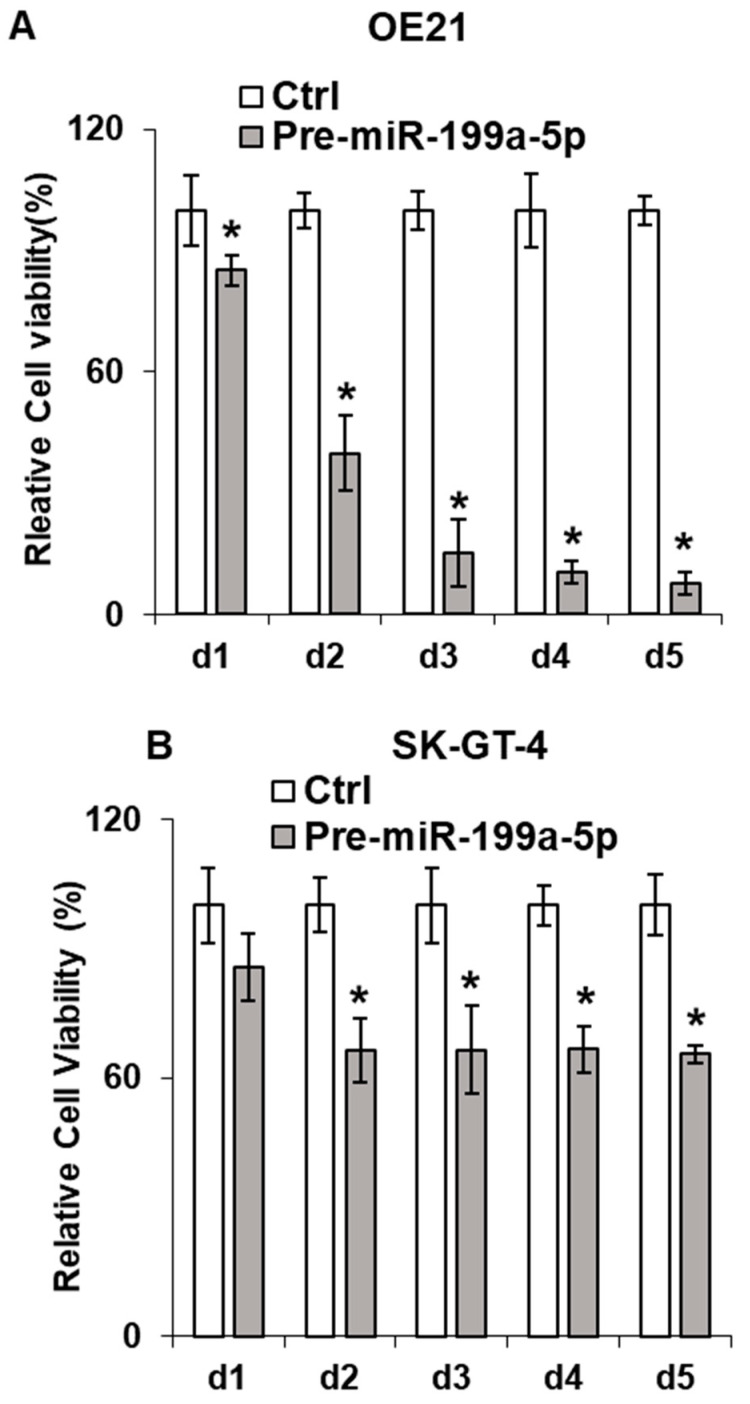

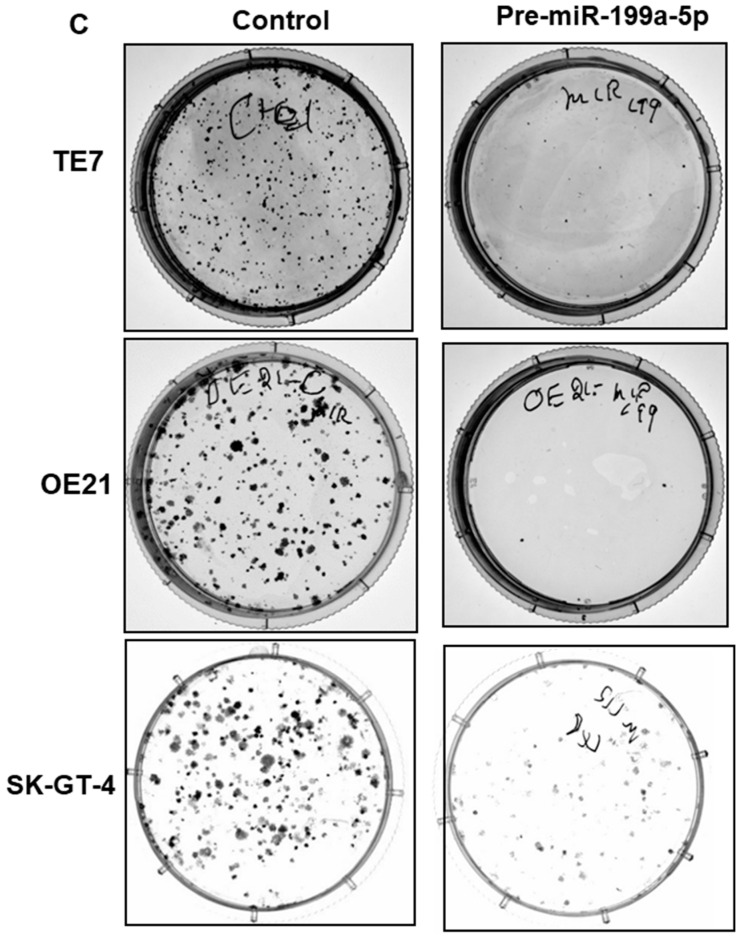
Effect of miR-199a-5p on esophageal cancer cell proliferation. Cell viability assays (MTT assay) in (**A**) OE21 and (**B**) SK-GT4 cells. Cells were transfected for the indicated time points with pre-miR-199a-5p (50 nM) and changes in viability were measured using 3-(4,5-Dimethylthiazol-2-yl)-2,5-diphenyltetrazolium bromide (MTT) for each time point. Bar diagram shows % cell growth. The mean absorbance for control cells not transfected with the pre-miR-199a-5p was considered as 100%. Mean ± SD of three independent experiments. (**C**) Colony formation assay. Following transfection with miR-199a-5p (50 nM), cells were harvested at 48 h and 2000 cells were reseeded in 60 mm dishes for the control and transfected samples and grown for 14 days. Colonies were stained with crystal violet. * represents statistical significance.

Given the association of Jun-B with cellular proliferation, we assessed proliferation in the esophageal cancer cells following overexpression of miR-199a-5p. As seen in Figure 4A,B, we observed a significant decrease in both OE21 and SK-GT-4 cellular proliferation following transfection of pre-miR-199a-5p as measured by MTT assay. Of note, a decrease in TE7 proliferation following overexpression of miR-199a-5p has been previously demonstrated [13]. Furthermore, a marked reduction is also observed in colony formation in all three-cell lines following 48 h of miR-199a-5p overexpression (Figure 4C).

**Figure 5:**
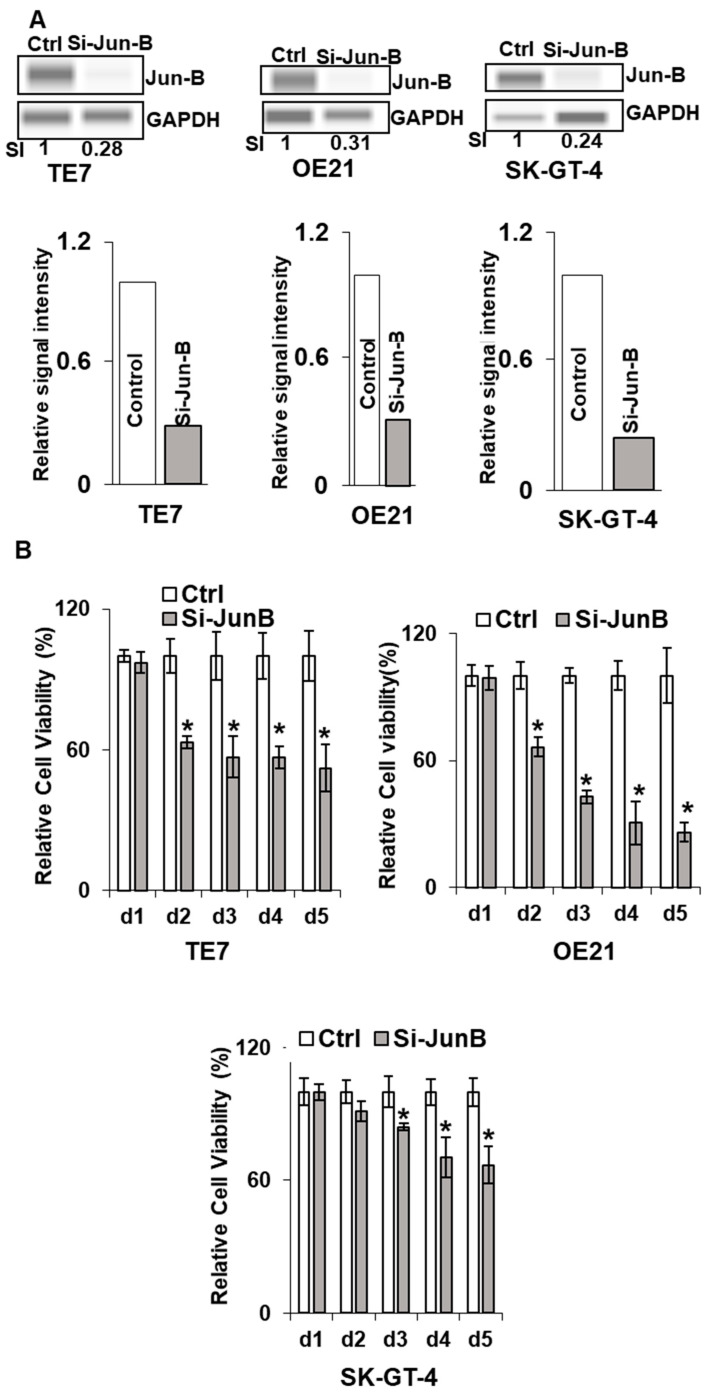

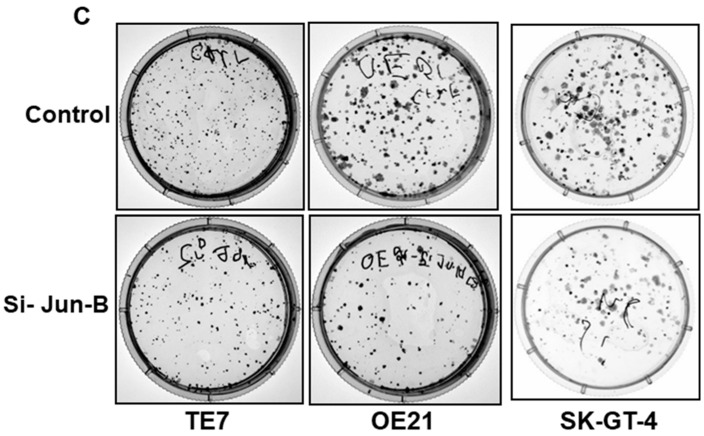
Effect of Jun-B silencing on esophageal cancer cells. (**A**) Changes in Jun-B protein expression levels in TE7 (left), OE21 (middle), and SK-GT-4 (right) cells following silencing with Jun-B siRNA (50 pmol). Whole-cell lysates were prepared for Western blotting and loading was assessed by GAPDH. (Bottom) Changes in signal intensity. Relative signal intensity of Jun-B in cells mentioned above, TE7 (left), OE21 (middle), and Sk-GT-4 (right). Jun-B intensity was normalized with GAPDH intensity and relative levels were measured compared to the control for each cell line, as explained in Figure 1E using compass software for JESS automated Western System. The signal intensity for the control is set as 1. Relative intensity in TE7, OE21, and SK-GT-4 cells with JunB silencing is 0.28, 0.31, and 0.24, respectively. (**B**) Cell viability assays (MTT assay) in TE7 (top left), OE21 (top right), and SK-GT4 cells (bottom). MTT assay was conducted as explained in Figure 4. (**C**) Colony formation assay in same cell lines after silencing Jun-B for 48 h. The assay was performed as explained above in Figure 4C. The uncropped blots and molecular weight markers are shown in Supplementary Figure S3. The * indicates statistical significance.

In order to determine if these observed decreases in cellular proliferation following the overexpression of miR-199a-5p were mediated by decreased Jun-B expression, these experiments were repeated following transfection of Jun-B specific siRNA. As seen in Figure 5A, transfection of Jun-B siRNA for 48 h resulted in a strong reduction in Jun-B protein expression in all three cell lines. To a similar degree as that observed following miR-199a-5p overexpression, the transfection of Jun-B siRNA resulted in a marked decrease in proliferation in all three cell lines as assessed by an MTT assay (Figure 5B). A similar reduction in colony formation was also seen in all three cell lines when assessed after 48 h of exposure to Jun-B siRNA (Figure 5C).

**Figure 6:**
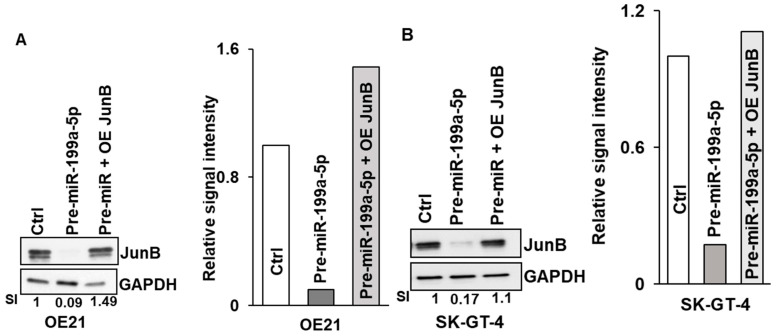

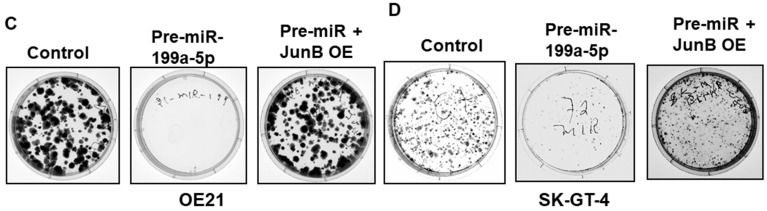
Effect of Jun-B overexpression on esophageal cancer cells. (**A**, **B**) Expression of Jun-B protein was measured in OE21 (**A**) and SK-GT-4 (**B**) cells, following overexpression of pre-miR-199a-5p (50 nM) (middle lane) or pre-miR-199a-5p followed 48 h later by 2.5 μg of Jun-B expression plasmid (last lane). Cells were harvested 72 h after the initial miR-199a-5p transfection. Jun-B levels in OE21 (**A**) and in SK-GT-4 (**B**) cells were measured by immunoblot and compared to control miR transfection; relative signal intensity was shown as a bar diagram and the ratio of intensity compared to the control is indicated at the bottom of the blots. (**C**, **D**) Colony formation assay in OE21 (**C**) and SK-GT-4 (**D**) cells was performed in the samples explained for immunoblot. The uncropped blots and molecular weight markers are shown in Supplementary Figure S4.

To further confirm that the observed reduction in cellular proliferation following overexpression of miR-199a-5p was mediated by the downregulation of Jun-B, rescue experiments were performed. As seen in Figure 6A,B, the transfection of OE21 and SK-GT-4 cells with a Jun-B expression plasmid following overexpression of miR-199a-5p results in restoration of Jun-B to baseline levels in these cells. Importantly, the restoration of Jun-B in these cells following miR-199a-5p overexpression results in an increase in colony formation back to control levels (Figure 6C,D).

### 3.5. Overexpression of miR-199a-5p Reduces AP-1 Promoter Activity

**Figure 7:**
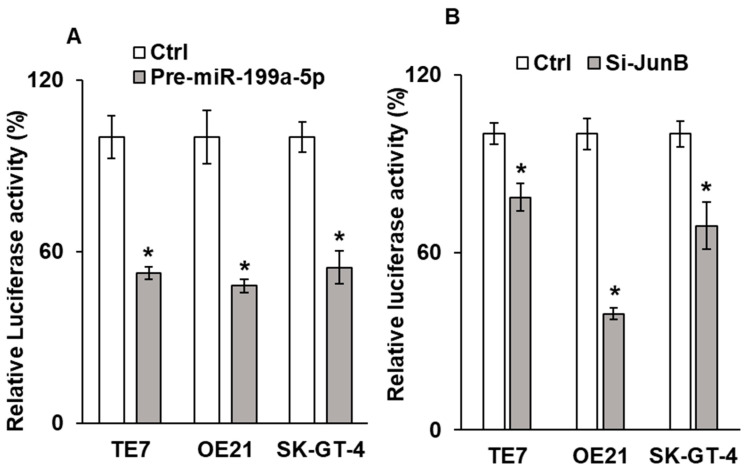
Effect of miR-199a-5p on AP1 promoter activity. Changes in AP1 promoter activity in TE7, OE21, and SK-GT-4 cells following (**A**) over expression of pre-miR-199a-5p and (**B**) Jun-B siRNA. Cells were co-transfected with the AP1 promoter luciferase reporter construct and either pre-miR-199a-5p or Jun-B siRNA for 48 h. Renilla was used to normalize the luciferase activity. Control cells not transfected with either pre-miR-199a-5p or pre-Jun-B siRNA were considered as 100%. * means Statistically significant.

To better understand the mechanism by which cellular proliferation was reduced, we next assessed AP-1 promoter activity following either miR-199a-5p overexpression or silencing of Jun-B, as Jun-B is a critical component of the AP-1 promoter. As seen in Figure 7A,B, AP-1 promoter activity is significantly reduced following manipulation in all three cell lines.

## 4. Discussion

These data expand upon our original findings by demonstrating markedly reduced expression of miR-199a-5p in multiple esophageal adenocarcinoma cell lines in addition to that previously seen in squamous cancer cell lines, compared to esophageal epithelial cells. In addition, we show the downregulation of miR-199a-5p in human esophageal cancer specimens compared to the matched esophageal epithelium. Furthermore, we demonstrate increased expression of Jun-B in a subset of these esophageal cancer cell lines and human esophageal tumor specimens relative to their non-malignant controls. Mechanistically, we show that miR-199a-5p downregulates Jun-B expression in these esophageal cancer cells through direct interaction with its mRNA, resulting in decreased Jun-B mRNA stability. Finally, either forced expression of miR-199a-5p or silencing of Jun-B results in markedly reduced cellular proliferation, likely mediated, in part, by reduced AP-1 promoter activity. 

MiR-199a-5p has previously been shown to be downregulated in multiple malignancies where it functions as a tumor suppressor, frequently by inhibiting cellular proliferation, as we have seen in esophageal cancer cells. The specific targets of miR-199a-5p vary across different malignancies. In renal cell cancer (RCC) cells, proliferation was found to be significantly decreased in cells following overexpression of miR-199a-5p. In these cells, this effect was found to be due to the ability of miR-199a-5p to downregulate the expression of GSK-3β, a serine/threonine kinase involved in NFκB signaling [18]. In colorectal cancer (CRC) cells, miR-199a-5p has been shown to suppress cellular proliferation, as well as the migration and invasion capabilities of these cells, by inhibiting expression of ITGA3, a member of the integrin family [19]. As in our study, expression of miR-199a-5p was also found to be decreased in human CRC samples. Similarly, overexpression of miR-199a-5p inhibited cellular proliferation in hepatocellular carcinoma (HCC) cells by binding to the mRNA of CDC25a to reduce its expression [20]. In this study, the expression of miR-199a-5p was also shown to be decreased in human HCC samples. In non-small cell lung cancer (NSCLC) cells, miR-199a-5p was found to profoundly decrease cellular proliferation by decreasing the expression of A-kinase anchoring protein 1 (AKAP1) [21]. Interestingly, in order to understand why miR-199a-5p was downregulated in human NSCLC samples, the authors demonstrated a marked increase in the methylation levels of the miR-199a-5p promoter in human NSCLC samples compared to adjacent non-malignant pneumocytes. 

The role of miR-199a-5p is not well described in esophageal cancer, although analyses of its function are consistent with its role as a tumor suppressor. The expression of miR-199a-5p was found to be decreased in squamous cell cancer stem cells [22]. In these cells, forced expression of miR-199a-5p resulted in the decreased proliferation and transcription of stemness marker genes. This effect was mediated by reduced expression of the miR-199a-5p target, Sirt1, which was required for intracellular proteolysis of CD44. This reduction in Sirt1 expression also impaired the nuclear translocation of the CD44 intracellular domain. A recent elegant study detailed how miR-199a-5p levels are reduced in esophageal cancer cells as part of the cellular response to radiation therapy [23]. In this study, the lnc-RNA NORAD was upregulated in esophageal cancer cells in response to DNA damage following exposure to radiation. NORAD was shown to sequester the RNA-binding protein PUM1 through a direct binding interaction, thus inhibiting the processing of pri-miR-199a1 to mature miR-199a-5p. The resulting downregulation of miR-199a-5p resulted in increased expression of its target, EEPD1, which is an important regulator of the repair of stressed replication forks to allow DNA repair and cell survival. 

Our identification of Jun-B as a target of miR-199a-5p enhances our understanding of the role and regulation of Jun-B by miRs in malignancy. Jun-B has been shown to mediate both tumor-suppressive and oncogenic roles in different malignancies. Based on its antagonistic effects on c-Jun-dependent transcription of cyclin D1, Jun-B has been demonstrated to be a negative regulator of cellular proliferation [24]. Furthermore, Jun-B has also been shown to enhance the transcription of p16^INK4a^, which inhibits phosphorylation of the Rb protein, thereby preventing the transition from G1 to S phase [25]. However, more recent studies describe the growth-promoting activities associated with Jun-B in certain cancers. Jun-B has been shown to be overexpressed in Hodgkin’s lymphoma and anaplastic large cell lymphomas [26,27]. Using the TCGA data set, Pei and colleagues have shown that Jun-B is upregulated in non-small cell lung cancer patients and is associated with decreased overall survival [28]. Although data on the oncogenic role of Jun-B in esophageal cancer are sparse, an analysis of the TCGA dataset indicated a statistically significant decrease in the survival of esophageal adenocarcinoma patients with increased Jun-B expression [29]. 

Data on the regulation of Jun-B by miRs in other cancers are sparse. Although miR-199a-5p has previously been shown to downregulate Jun-B expression in cardiac myocytes to promote apoptosis, this relationship has not previously been demonstrated in any malignancy [30]. MiR-149 has been shown to exert an oncogenic role in T-cell acute lymphoblastic leukemia by downregulating the expression of Jun-B in these cells, leading to enhanced cellular proliferation and decreased apoptosis [31]. Similarly, in pancreatic cancer, exosomal miR-95 functioned as a tumor promoter by binding to and decreasing the expression of Jun-B to promote cellular proliferation and invasion [32]. Conversely, in esophageal squamous cell cancer lines, miR-615-5p overexpression was found to result in the downregulation of Jun-B, resulting in decreased cellular proliferation and migration [33]. 

The results of the current study, which demonstrates direct regulation of Jun-B by miR-199a-5p, add to our previous findings by providing additional evidence that miR-199a-5p is an important regulator of cellular proliferation in esophageal cancer cells through its multiple effects on AP-1 promoter activity. This important transcription factor, composed of c-JUN, Jun-B, and Fos family proteins, has been shown to be involved in the regulation of diffuse cellular processes including proliferation, differentiation, migration, and apoptosis [14]. In our previous study, we found that miR-199a-5p bound and reduced the expression of MAP3K11 in esophageal cancer cells [13]. In the MAP kinase signaling pathway, activated MAP3K11 phosphorylates and activates MAP2K, which then activates a wide range of transcription factors, including JNK and p38 [34]. JNK is an important activator of c-Jun. We showed that the reduction in MAP3K11 expression following overexpression of miR-199a-5p resulted in decreased levels of phosphorylated c-Jun, leading to impaired transcription of cyclin D1 and decreased cellular proliferation. Our current results show that miR-199a-5p can bind to Jun-B mRNA, resulting in decreased Jun-B mRNA and expression. The resulting downregulation of Jun-B results in reduced AP-1 promoter activity and decreased cellular proliferation. It is important to note, however, that Jun-B expression was not elevated in all cell lines with low miR-199a-5p levels or in all human esophageal tumor specimens, suggesting that additional mechanisms are involved in regulating Jun-B expression in these cells. 

By virtue of its effects to regulate cellular proliferation through the downregulation of two components of the AP-1 transcription factor, miR-199a-5p is emerging as a potential critical regulator in esophageal cancer cells. A similar role for miR-199a-5p is being demonstrated in ovarian cancer cells. The restoration of miR-199a-5p expression in ovarian cancer cells has been shown to increase sensitivity to cisplatin through targeting both mTOR and CD44, thereby reducing ovarian cancer stem cell levels [35,36]. Furthermore, miR-199a-5p was also shown to target IkappaB kinase-beta in ovarian cancer cells, resulting in increased sensitivity to TNF-α-induced apoptosis following forced expression of miR-199a-5p [37]. 

Studies such as these hold great potential for unlocking the ability of individual miRs to serve as both biomarkers and therapeutic targets for cancer patients. To date, specific miR expression signatures both in tumor tissue and in patient serum have been identified with prognostic and predictive capabilities [38,39,40,41]. As the age of RNA-based therapeutics dawns, the use of miRs to treat patients with mesothelioma and other solid malignancies have begun to be tested in phase 1 clinical trials [42,43]. Before testing the therapeutic efficacy of miR-199a-5p in esophageal cancer, future efforts will need to be directed at identifying additional targets of miR-199a-5p that regulate other critical oncogenic processes in esophageal cancer cells such as apoptosis, migration, and chemoresistance. Finally, pre-clinical testing of delivery by both systemic and intratumoral routes of administration will be critical to optimize the potential of miR-199a-5p-based therapies for esophageal cancer patients. 

## 5. Conclusions

We demonstrated that miR-199a-5p was downregulated in human esophageal cancer specimens and cell lines while Jun-B expression was upregulated in these cells. MiR-199a-5p negatively regulates Jun-B by reducing the stability of its mRNA. MiR-199a-5p acts as a tumor suppressor in esophageal cancer cells through repression of Jun-B, resulting in decreased cellular proliferation.

## Figures and Tables

**Table 1:** Jun-B primer sequences used for cloning. (Bold sequences in the table indicate respective restriction enzyme sequences).

SN	Name	Sequence	Region	Cutting Site
1	pmiR-GLO-Jun-B-3′UTR-F	**GAGCTC** CCAAGAGCGCATCAAAGTGG	1081–1716	SacI
2	pmiR-GLO-Jun-B-3′UTR-R	**TCTAGA** TTCCACAGTACGGTGCAGAG	1081–1716	XbaI
3	pmiR-GLO-Jun-B-CR-F	**GAGCTC** CCCTTCTACCACGACGACTC	308–839	SacI
4	pmiR-GLO-Jun-B-CR-R	**TCTAGA** GGTTGGTGTAAACGGGAGGT	308–839	XbaI
5	pmiR-GLO-Jun-B-3′UTR-Del-F	GCGCCGCAAACCCTCCGGCCCTCC	1081–1716	N/A
6	pmiR-GLO-Jun-B-3′UTR-Del-R	GGAGGGCCGGAGGGTTTGCGGCGC	1081–1716	N/A

## Data Availability

The data supporting the finding of this study are available within the article and are available from the corresponding authors upon request.

## Supplementary Materials

The following supporting information can be downloaded at: https://www.mdpi.com/article/10.3390/cancers15194811/s1, Figure S1: Original blot for Figure 1D. Figure S2: Original blot for Figure 2B. Figure S3: Original automated blot for Figure 5A. Figure S4: Original blot for Figure 6A,B.
