# Supplementary material for: MiR-199a-5p Decreases Esophageal Cancer Cell Proliferation Partially through Repression of Jun-B
Source: Cancers (Basel). 2023 Sep 30;15(19):4811. doi: 10.3390/cancers15194811 (PMC10571772; doi:10.3390/cancers15194811)
Supplement: Supplementary file 1 [file cancers-15-04811-s001.zip › Fig-S1-Original blot for figure 1D.pdf]

Fig. S1 **Full unedited gel for figure 1D.**

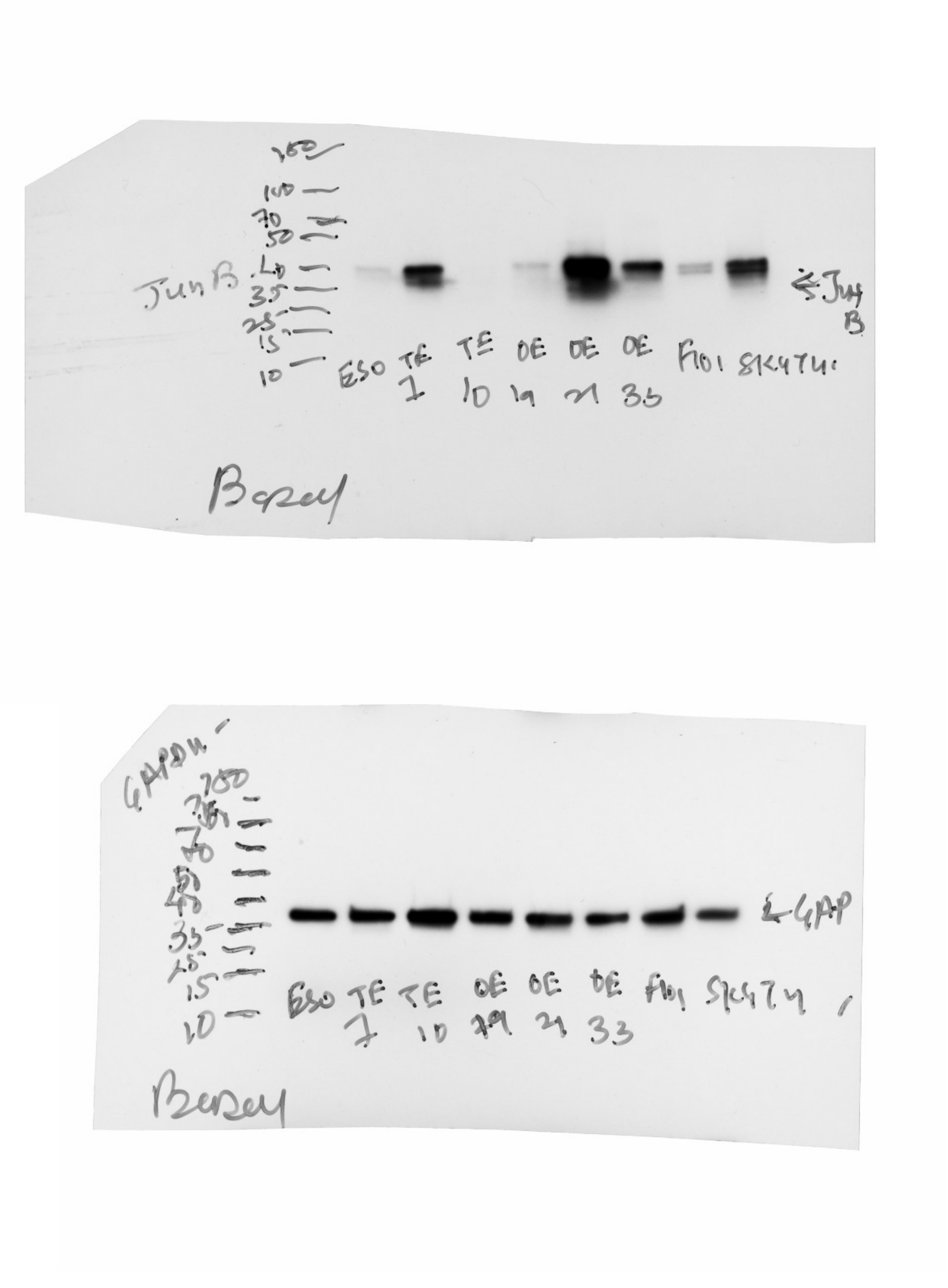

Fig S1. Original blot for figure 1D. Endogenous JunB protein expression levels in the human esophageal cell lines (top). GAPDH was used as a loading control (bottom)
