# Supplementary material for: MiR-199a-5p Decreases Esophageal Cancer Cell Proliferation Partially through Repression of Jun-B
Source: Cancers (Basel). 2023 Sep 30;15(19):4811. doi: 10.3390/cancers15194811 (PMC10571772; doi:10.3390/cancers15194811)
Supplement: Supplementary file 1 [file cancers-15-04811-s001.zip › Fig-S2A-Original blot for figure 2B-TE7.pdf]

Fig.S2A

Full unedited gel for figure 2B (TE7)

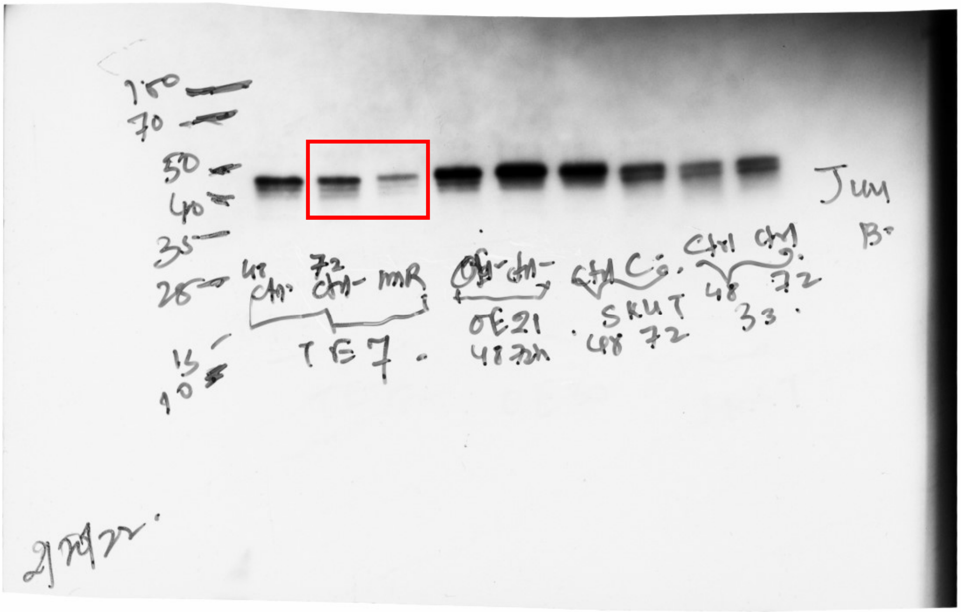

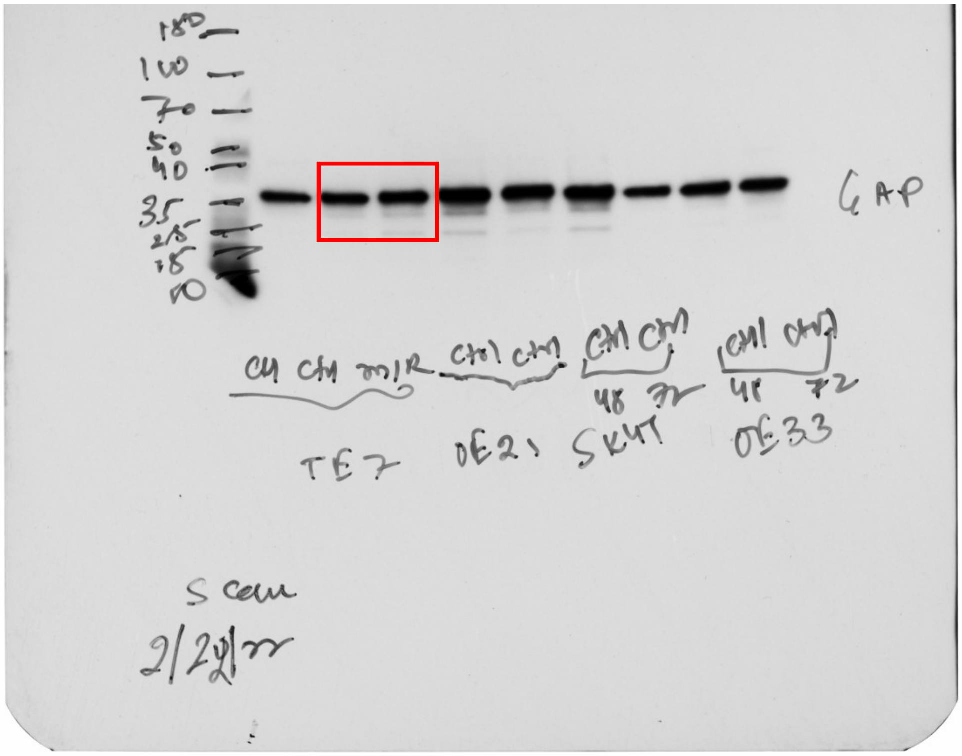

Fig.S2A. Original blot for figure 2B (TE7, left panel) Changes in JunB protein expression after overexpressing miR-199a-5p in TE7 cells (top). Protein loading was assessed by GAPDH (bottom)
