# Supplementary material for: MiR-199a-5p Decreases Esophageal Cancer Cell Proliferation Partially through Repression of Jun-B
Source: Cancers (Basel). 2023 Sep 30;15(19):4811. doi: 10.3390/cancers15194811 (PMC10571772; doi:10.3390/cancers15194811)
Supplement: Supplementary file 1 [file cancers-15-04811-s001.zip › Fig-S2B-Original blot for figure 2B-OE21-SKGT.pdf]

Fig.S2B

Full unedited gel for figure 2B (OE21 and SK-GT-4)

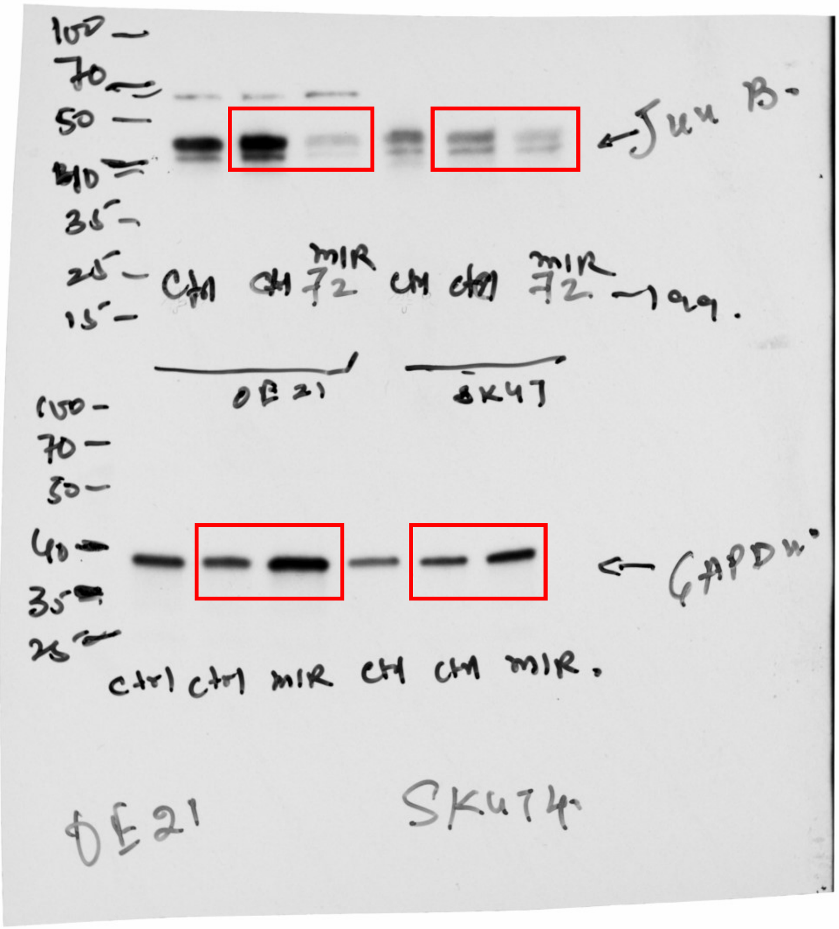

Fig.S2B. Original blot for figure 2B (OE21, middle panel and SK-GT-4, right panel). Changes in JunB protein expression following over expression of pre-miR-199a-5p in OE21and SK-GT-4 cells (top). Protein loading was assessed by GAPDH (bottom).
