# Supplementary material for: MiR-199a-5p Decreases Esophageal Cancer Cell Proliferation Partially through Repression of Jun-B
Source: Cancers (Basel). 2023 Sep 30;15(19):4811. doi: 10.3390/cancers15194811 (PMC10571772; doi:10.3390/cancers15194811)
Supplement: Supplementary file 1 [file cancers-15-04811-s001.zip › Fig-S3-Original automated blot for figure 5A.pdf]

Fig. S3

Full unedited gel for figure 5A

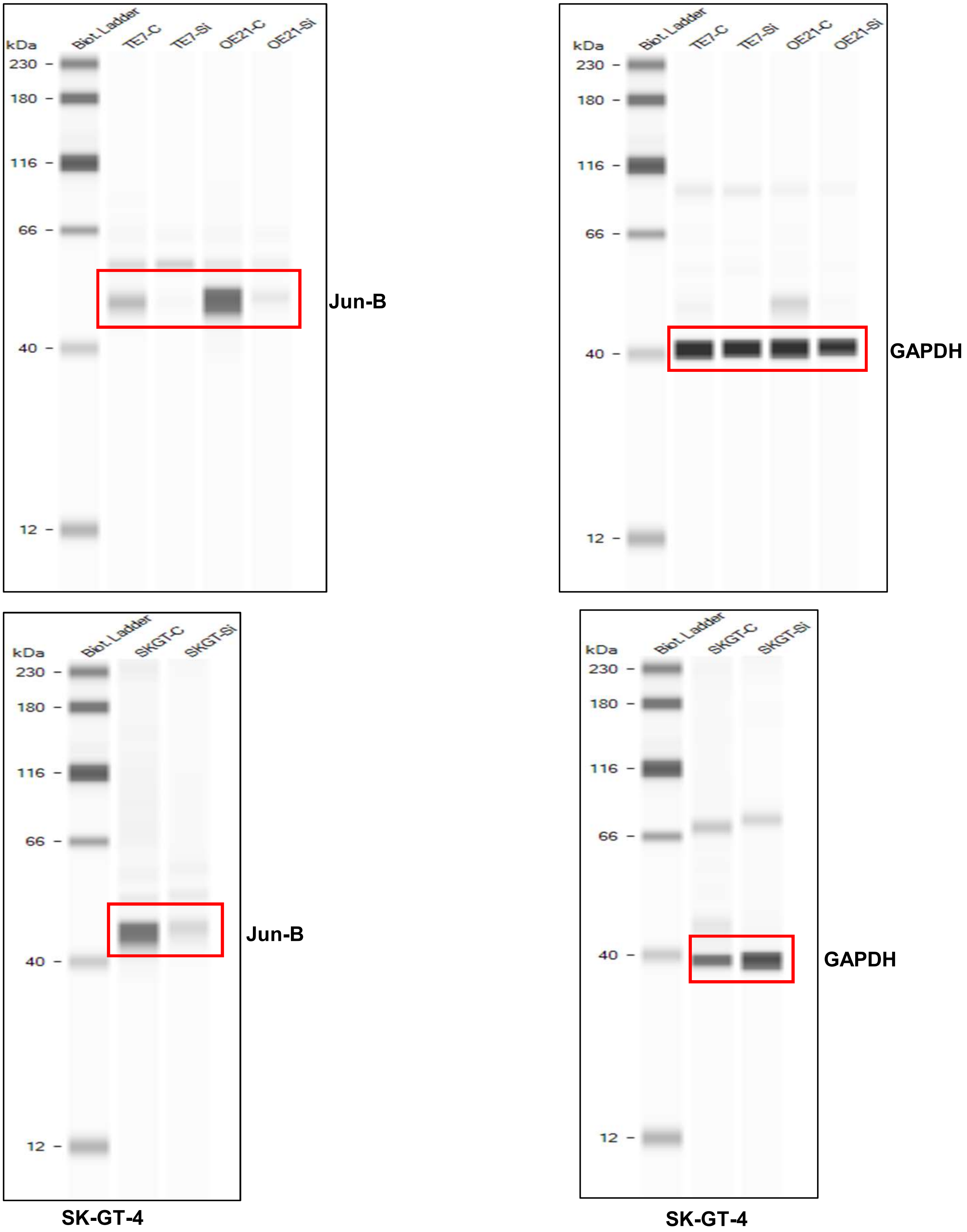

Fig. S3. Original blot for figure 5A . Changes in JunB protein expression following silencing with si-JunB RNA, in TE7 and OE21 (Top) and SK-GT-4 cells (bottom). Protein loading was assessed by GAPDH. These images were directly exported from the automatic Jess western system using Compass software. First lane in all the images represents biotin molecular weight ladder.
