# Supplementary material for: MiR-199a-5p Decreases Esophageal Cancer Cell Proliferation Partially through Repression of Jun-B
Source: Cancers (Basel). 2023 Sep 30;15(19):4811. doi: 10.3390/cancers15194811 (PMC10571772; doi:10.3390/cancers15194811)
Supplement: Supplementary file 1 [file cancers-15-04811-s001.zip › Fig-S4A-Original blot for figure 6A-OE21.pdf]

Fig. S4A

Full unedited gel for figure 6A

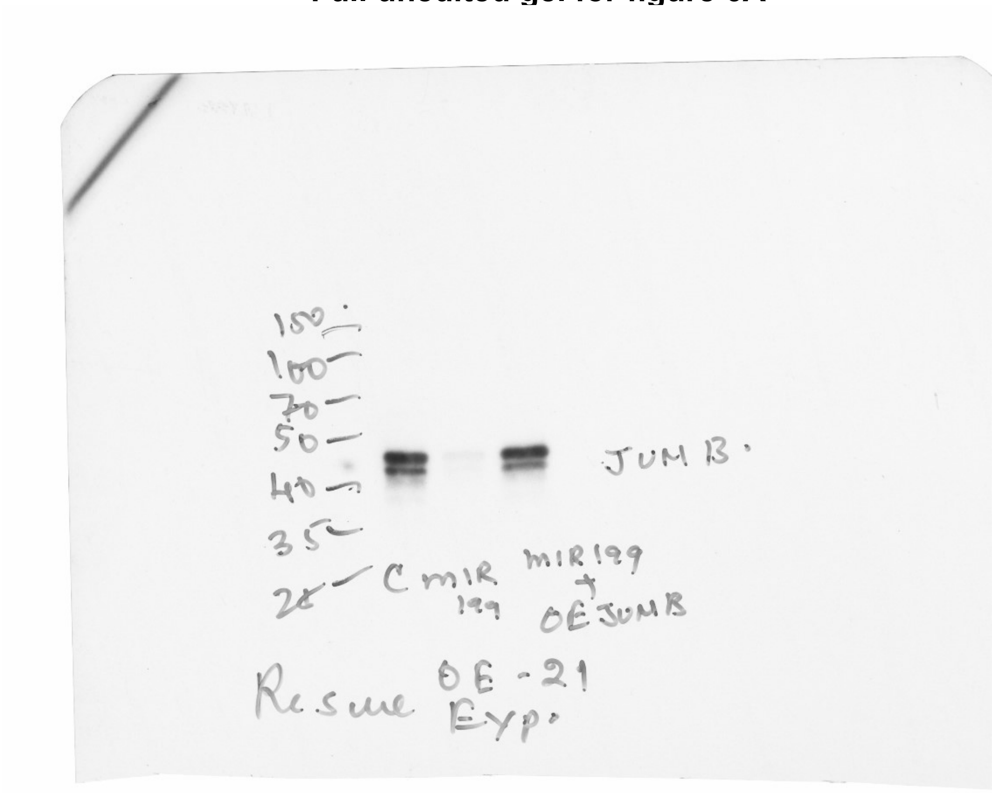

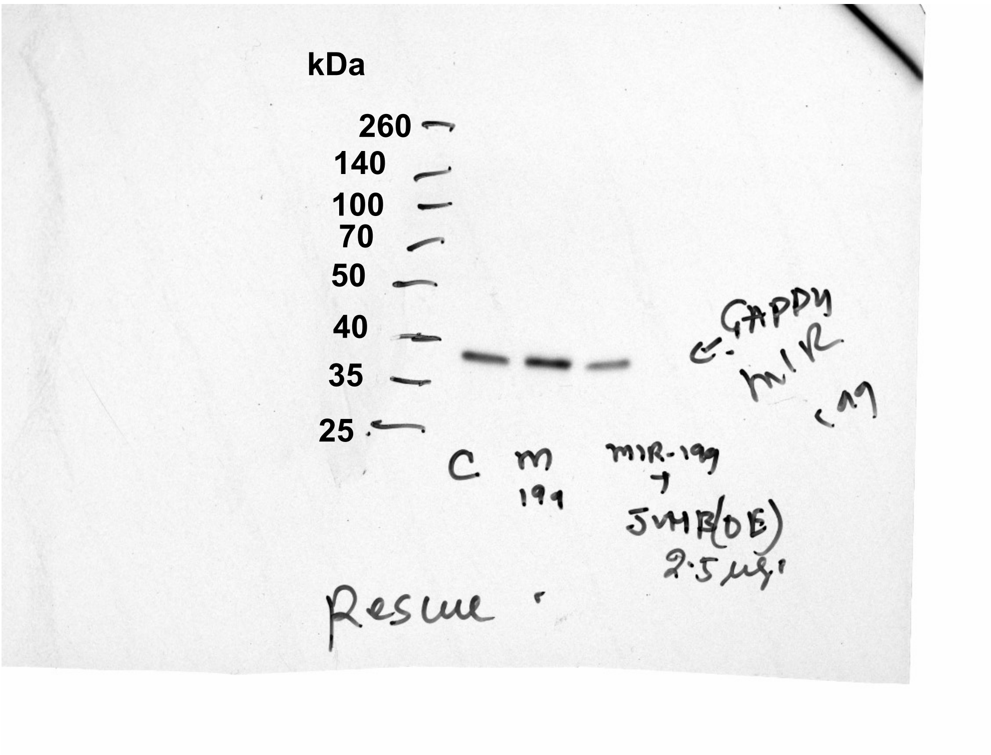

Fig. S4A. Original blot for figure 6A . Changes in JunB protein expression (top), in control (first lane), after overexpressing pre-miR-199a-5p alone (middle lane) and co expressing with JunB plasmid (last lane). Protein loading was assessed by GAPDH (bottom).
